# Knowledge, Attitudes and Practices Survey of Recombinant Zoster Vaccine among Cardiologists and Cardiac Nurses in Italy

**DOI:** 10.3390/medicina60010093

**Published:** 2024-01-03

**Authors:** Domenico Ponticelli, Ippazio Cosimo Antonazzo, Lorenzo Losa, Anna Zampella, Fabio Di Marino, Gaetano Mottola, Mara Noemi Fede, Fortuna Gallucci, Roberto Magliuolo, Antonio Rainone, Carmine Del Giudice, Antonella Arcari, Pietro Ferrara

**Affiliations:** 1 Clinica Montevergine SpA, 83013 Mercogliano, Italy; 2 Center for Public Health Research, University of Milan–Bicocca, 20900 Monza, Italy; 3 Laboratory of Public Health, IRCCS Istituto Auxologico Italiano, 20165 Milan, Italy; 4 Independent Researcher, 81030 Lusciano, Italy

**Keywords:** cardiology, herpes zoster, recombinant zoster vaccine, vaccine literacy

## Abstract

*Background and Objectives*: Cardiac patients are particularly at risk of herpes zoster (HZ), which is associated with a higher risk of major cardiovascular events. This research aimed to analyze the knowledge, attitudes and practices towards recombinant zoster vaccine (RZV) among cardiac healthcare professionals (HPs). *Materials and Methods*: A cross-sectional survey was conducted in a cardiological hospital in Italy. Multivariate regression models were built to identify factors associated with the outcomes of interest. *Results*: The response rate was 78.2% (154/197). Overall, age > 50 years and immunosuppression were recognized as risk factors for HZ by 38.3% and 75.3% of respondents, respectively. Regarding RZV, 29.1% of the HPs correctly responded about its schedule and 57.6% about the possibility of administration in immunocompromised individuals. This knowledge was significantly higher in HPs with a higher educational level (odds ratio (OR) = 4.42; 95%CI 1.70–11.47), in those who knew that HZ could cause postherpetic neuralgia (OR = 2.56; 95%CI 1.05–6.25) or major cardiovascular events (OR = 4.23; 95%CI 1.50–11.91), in those who had participated in professional updates on vaccinations (OR = 3.86; 95%CI 1.51–9.87) and in those who stated the need for further information about the RZV (OR = 6.43; 95%CI 1.42–29.98). Younger HPs (coefficient (*β*) = −0.02; 95%CI −0.04–−0.01), those with a positive attitude toward RZV safety (*β* = 2.92; 95%CI 2.49–3.36) and those who had previously cared for patients with HZ (*β* = 0.45; 95%CI 0.03–0.88) reported a more positive attitude toward RZV effectiveness. The practice of recommending vaccination was more prevalent in younger HPs (OR = 0.94; 95%CI 0.89–0.99), in those who had a master’s degree or higher education (OR = 7.21; 95%CI 1.44–36.08), in those with more positive attitudes toward RZV effectiveness (OR = 7.17; 95%CI 1.71–30.03) and in HPs who had already recommended the vaccine to patients in the past (OR = 4.03; 95%CI 1.08–14.96). *Conclusions*: Despite being a single-center study, our research brings attention to factors that currently impact cardiac HPs’ approaches to RZV. The findings indicate potential measures to enhance HPs’ awareness and practices, ultimately aiming to improve vaccination adherence and reduce the burden associated with HZ.

## 1. Introduction

Herpes zoster (HZ), or shingles, caused by latent varicella zoster virus (VZV), is a syndrome occurring when immunity to VZV declines due to age or immunosuppression. This reactivation causes a painful, unilateral and vesicular rash, following the viral spread from a dorsal root or cranial nerve ganglion to the corresponding dermatome [1,2]. HZ can be complicated by chronic pain (postherpetic neuralgia (PHN)) and other sequelae, such as neurological and ophthalmological disorders (e.g., meningoencephalitis, myelitis, cranial nerve palsies, vasculopathy, keratitis and loss of vision) [3].

Significant variability in HZ incidence has been observed across geographical areas: 6.6–9.0 cases per 1000 person-years has been estimated for North America, 5.2–10.9 for Europe and 10.9 for the Asia-Pacific region [4,5,6]. Variability has been also observed between sexes and age classes: higher incidence in females compared with males and in those 50 years or older compared with younger individuals. Among the various populations at an increased risk of developing herpes zoster (HZ), it is essential to mention individuals with primary and secondary immunosuppression (such as people living with human immunodeficiency virus infection/acquired immune deficiency syndrome, and patients taking immunosuppressive medications, like after an organ transplant or for other conditions), individuals with chronic diseases (such as diabetes and certain autoimmune diseases) and cancer patients [7].

Of note, the HZ burden in individuals with cardiovascular diseases is particularly significant. These patients inherently have an increased risk of HZ, recurrences and PHN due to advanced age and the frequent presence of comorbidities. Several studies have highlighted the elevated incidence of herpes zoster in patients with cardiac diseases, with estimates indicating a potential doubled risk in this population [8,9,10,11]. More recently, a pivotal meta-analysis observed a pooled risk increase in cardiovascular conditions of about 34% [7]. Therefore, this correlation warrants careful consideration concerning strategies for vaccination prevention against HZ [9]. Moreover, it has been observed that HZ is associated with an almost 30% higher risk of a major cardiovascular event, and this elevated risk persists for 12 years and more following HZ [12,13,14,15].

Vaccination is, therefore, crucial to limit the impact associated with HZ. Currently, two vaccines against HZ are licensed: a first single dose live attenuated vaccine (ZLV), authorized in Italy in 2006, and a newer two-dose adjuvanted recombinant glycoprotein E (gE) subunit vaccine (RZV), available since 2021. The latest Italian National Vaccination Plan (Piano Nazionale della Prevenzione Vaccinale, PNPV) for 2023 states that shingles vaccination should be offered every year to individuals aged 65 and older, and to at-risk individuals (e.g., due to illness or states of primary or acquired immunosuppression) from 18 years of age, with one of the two vaccines, according to the guidelines [16]. However, vaccination coverage against HZ remains low, and one of the objectives of the PNVP is to achieve a coverage of at least 50% among individuals aged sixty-five and older [16].

Among the strategies to increase vaccination are healthcare professional (HP) behaviors, which directly impact and can be effective in increasing uptake [17]. Indeed, they play an essential role in improving patient adherence to vaccination [18,19,20]. This involves not only medical staff but also other HPs. It is known that, among others, nurses play a key role in health promotion and health education, such as providing relevant information to address the reasons for not vaccinating [21,22].

However, the currently available literature has only lightly investigated the knowledge and attitudes of healthcare personnel towards RZV, and literature gaps still exist on the topic at hand [23,24,25]. In particular, nothing has been published on the knowledge, attitudes and practices of cardiac HPs on this topic, despite the greater clinical benefit of vaccinations for patients with cardiovascular conditions, as mentioned earlier. Similarly, studies investigating HPs’ awareness in Italy of HZ and vaccination strategies for at-risk populations are very limited [26]. To the best of our knowledge, there are no studies characterizing attitudes and knowledge towards immunization practices with the RZV vaccine.

## 2. Materials and Methods

### 2.1. Study Design

With the aim of evaluating the knowledge, attitudes and practices of vaccination among cardiologists, non-cardiologist physicians and cardiac nurses, a cross-sectional survey was conducted in a cardiological hospital located in southern Italy. The facility specializes in the diagnosis, treatment and care of patients with heart-related conditions and cardiovascular diseases. It is equipped with specialized HPs, advanced diagnostic tools, and treatment options specifically tailored for cardiac care.

A cross-sectional questionnaire was conducted in November 2023 among all the medical doctors and nurses who provide direct patient care in the hospital. HPs were extended an invitation to engage in this study via an online questionnaire dispatched to their professional email addresses by the hospital administration, using a tool specifically developed by the Information Technology unit of the venue hospital. Comprehensive information regarding the research goals, the guarantee of anonymity in data utilization and the freedom to withdraw from the study at any point was communicated to the participants. Involvement was entirely voluntary, without any incentives.

### 2.2. Survey Instrument

To assess the knowledge, attitudes and practices of cardiac HPs towards RZV, data were collected using a three-part self-administered questionnaire that comprised a series of questions adapted to the Italian context from the existing literature [23,24,25]. Part one of the questionnaire gathered information on HPs’ demographic and professional characteristics, such as gender, age, educational level, professional role, area of work (i.e., the hospital unit), experience of managing patients with HZ and previous professional updates on vaccinations. Part two was designed to collect information on knowledge, attitudes and practices about RZV. Specifically, the participants were asked for information regarding their knowledge about major risk factors and complications associated with HZ, knowledge of the vaccination schedule (i.e., number of doses and timing), knowledge that the vaccine is included in the PNPV and that there is the possibility of administration in immunocompromised individuals, attitudes toward RZV safety and effectiveness, and practices to recommend HZ vaccination to patients. Finally, the last part of the questionnaire investigated HPs’ attitudes toward the need for further information about the RZV, as well as their perception that patients are being offered too many vaccinations or may not accept the proposal for an ‘additional’ vaccine. The questionnaire underwent pre-testing and piloting with a convenience sample of 10 HPs resembling the study population. Based on the respondents’ suggestions, minor revisions were made, involving adjustments to the wording and format of certain questionnaire items.

### 2.3. Statistical Analysis

The statistical analysis was conducted using Stata version 18 statistical software [27]. It consisted of descriptive and inferential analyses. The statistical estimates were shown as frequencies and percentages for categorical variables and mean and standard deviation (SD) for continuous variables.

Adjusted regression models were used to assess the association between predictors and the outcomes of interest (i.e., knowledge, attitudes and practices towards RZV). Three separate multivariate models were performed to determine independent characteristics associated with these outcomes: knowledge about the possibility of RZV administration in immunocompromised individuals (Model 1); attitude toward vaccine effectiveness (measured on an ordinal scale from 1 to 10; Model 2); practice of recommending HZ vaccination to patients (Model 3). In all models, the following explanatory independent variables were studied for inclusion: gender (female = 1; male = 0), age (continuous, in years), educational level (master’s degree or higher = 1; other = 0), professional role (physician = 1; nurse = 0), previous professional update on vaccinations (yes = 1; no = 0), experience in managing patients with HZ (yes = 1; no = 0), knowledge of HZ risk factors (e.g., age > 50 years: yes = 1; no = 0; presence of multiple chronic conditions: yes = 1; no = 0; etc.), awareness of major HZ consequences and complications (e.g., PHN: yes = 1; no = 0; HZ recurrence: yes = 1; no = 0; major cardiovascular events: yes = 1; no = 0), knowledge that HZ vaccines are included in the PNPV (yes = 1; no = 0) and need for further information about the RZV (yes = 1; no = 0). To investigate how knowledge affects attitudes and practices, as well as the impact of attitudes on practices, the models were constructed using a step-by-step approach, wherein the outcomes of the preceding models were subsequently studied for inclusion as explanatory variables in the subsequent models. The following variables were also included in Models 2 and 3: correct knowledge of the possibility of RZV administration in immunocompromised individuals (yes = 1; no = 0), attitude toward RZV safety (high = 1; middle to low = 0) and having previously ever recommended or suggested HZ vaccination to at-risk patients (yes = 1; no = 0). Variables such as attitude toward RZV effectiveness (high = 1; middle to low = 0), perception that patients are being offered too many vaccinations (agree = 1; disagree = 0) and idea that they may not accept the proposal for an ‘additional’ vaccine (agree = 1; disagree = 0) were also included in Model 3.

To facilitate our analysis and interpretation, some variables measured on ordinal scales were dichotomized before model building. According to the stepwise method for multivariate analysis, variables with a *p*-value < 0.25 in multivariate analysis were considered for inclusion in the final logistic and linear regression models. Results of logistic regression (Models 1 and 3) were reported as odds ratios (ORs) and 95% confidence intervals (CIs), while standardized regression coefficients (*β*) and 95%CIs were presented in the linear regression model (Model 2). The significant statistical level for *p*-values was set at 0.05.

## 3. Results

A total of 197 HPs were invited to fill out the questionnaire, and 154 agreed to participate in this study, giving us an overall response rate of 78.2%. The main demographic and professional characteristics of the study population are listed in Table 1. Fewer than two-thirds of the participants were women (62.3%), there was a mean age of 45.7 years and the participants were mainly nurses (64.3%). Approximately 40% of the participants had previously received a professional update on vaccinations or had experience in managing patients with HZ.

The distribution of responses concerning HPs’ knowledge, attitudes, and practices regarding RZV is presented in Table 2. In terms of their knowledge on the risk factors associated with HZ, except for immunosuppression (identified by 75% of respondents), knowledge of other factors was reported by fewer than 40% of HPs. Regarding complications or consequences of HZ, 53.2% knew it could cause PHN, 42.2% that it could recur and 35.7% that it could cause major cardiovascular events. Knowledge of the inclusion of HZ vaccines in PNPV was limited to 10.9% of the respondents.

Regarding RZV, 29.1% of the HPs knew the correct vaccination schedule, and 57.6% knew that the vaccine can be administered in patients with immunosuppressive conditions.

The findings from the multivariable logistic regression analysis indicated that the odds of the latter knowledge were higher in HPs with a master’s degree or higher as their educational level (OR = 4.42; 95%CI 1.70–11.47), in those who knew that HZ can cause major cardiovascular events (OR = 4.23; 95%CI 1.50–11.91) or PHN (OR = 2.56; 95%CI 1.05–6.25), in those who had received a professional update on vaccinations (OR = 3.86; 95%CI 1.51–9.87) and in those who stated the need for further information about RZV (OR = 6.43; 95%CI 1.42–29.98) (Model 1 in Table 3).

The degree of positivity of attitudes toward RZV safety and effectiveness was moderate to high, with 58.7% and 55.8%, respectively, of the participants scoring more than 7 on a scale from 1 to 10. Results of the multivariable linear regression model indicated a more positive attitude toward the RZV effectiveness in younger HPs (*β* = −0.02; 95%CI −0.04–−0.01), in those with a positive attitude toward RZV safety (*β* = 2.92; 95%CI 2.49–3.36) and in HPs who had previously cared for patients with HZ (*β* = 0.45; 95%CI 0.03–0.88).

The practice of having previously suggested HZ vaccination to patients was reported in 46.3% of the HPs, while willingness to recommend it in the future was declared by 78.9% of the respondents. The multivariate regression analysis found that the latter was more prevalent in younger HPs (OR = 0.94; 95%CI 0.89–0.99), in those who had a master’s degree or higher education (OR = 7.21; 95%CI 1.44–36.08), in those with more positive attitudes toward RZV effectiveness (OR = 7.17; 95%CI 1.71–30.03) and in HPs who had already recommended the vaccine to patients in the past (OR = 4.03; 95%CI 1.08–14.96).

**Figure 1:**
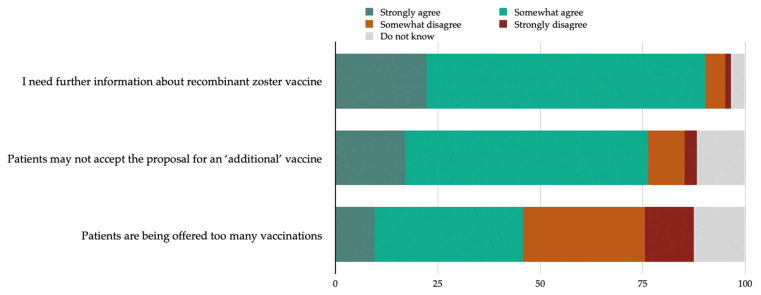
Health professionals’ attitudes regarding the need for more information about RZV and the delivery of RZV to adult patients.

Lastly, the vast majority (90.3%) of the HPs stated the need for more information about RZV, while 45.9% reported a belief that patients are being offered too many vaccinations, and 76.3% that patients may not accept the proposal for an ‘additional’ vaccine (Figure 1).

## 4. Discussion

This study has produced interesting results regarding the knowledge, attitudes and practices in a sample of Italian cardiologists and cardiac nurses towards RZV, two years after its licensing in Italy. To our knowledge, this represents the first assessment of cardiac HPs on the subject of RZV. The findings revealed that, although approximately 40% of interviewees reported having experience in managing patients with HZ, their level of awareness regarding potential risk factors associated with HZ was unsatisfactorily low. Of note, only about 40% of responders knew that HZ could recur and even fewer were aware of the potential cardiovascular consequences of an HZ infection. It was surprising that fewer than 50% of the interviewees reported having knowledge of the vaccination schedule. Meanwhile, just over 50% knew that RZV can be administered to immunocompromised individuals, a group of patients particularly at risk of HZ and its consequences.

The multivariate analyses suggested that younger HPs were more likely to show a positive attitude toward the safety and effectiveness of RZV, and they were the most prone to recommending the vaccination against HZ. In the previous literature, it was observed that a younger age among healthcare workers was a driver for increased vaccine knowledge and ability to recommend the vaccines [19]. Like our findings, another significant driver was participation in specific training events, enhancing not only knowledge but also adherence to vaccination guideline recommendations. In general, our key findings are that those with a higher educational level and those with a younger age are more likely to be compliant with continuing medical education and receive updates about medical science; therefore, it is possible to speculate that they are less reluctant to recommend new vaccines to patients [28].

Almost all the interviewees reported the need for more information about RZV, even if more than half of the responders were concerned about the reluctance of patients to receive an additional vaccine. This is a well-known issue related to vaccination campaigns. In fact, appropriate HP-provided educational information about the safety and efficacy of vaccines might result in increasing coverage rates. In this context, considering the potential misleading information (i.e., adverse event occurrence) patients may have received about vaccines, the role of clinicians is paramount, especially when vaccines are recommended and not obligatory [19,29,30]. These remarks reveal the importance of patients receiving detailed information on potential risks associated with the use of vaccines as well as the beneficial effects of vaccination campaigns for patients and the community.

Another factor that impacts vaccination coverage rate is HPs’ beliefs. In our study, almost half of the interviewees thought that patients received too many vaccines. The possibility cannot be excluded that these HPs are less prone to offering non-obligatory vaccines, like RZV, than other HPs. This underlines the pivotal role of HPs in influencing patients’ willingness to receive a vaccine [31,32]. With regards to the HZ vaccine, a recent review indicated that specific HPs’ recommendations were positively associated with the patients’ willingness to undergo vaccination [33].

In brief, our study adds new information about HPs’ knowledge, attitudes and practices toward RVZ, which can be used when developing intervention strategies to increase HZ vaccination coverage and acceptance in at-risk patients. In fact, HZ vaccination uptake is relatively low worldwide, with only 24% of adults aged ≥ 50 years having been vaccinated in the USA and even lower coverage in other countries such as Australia, Canada and Turkey [34,35,36]. In the context of HZ, as for other vaccine-preventable diseases, patients’ beliefs, lack of information and education about vaccines’ benefit–safety profiles, as well as HPs’ viewpoints and attitudes toward vaccination may represent important determinants that can impact patients’ HZ vaccination willingness and consequently vaccination coverage [3,23,37,38,39]. Additionally, when considering the impact of outbreaks, and also the impact of the COVID-19 vaccination campaign, we can surmise that healthcare workers represent the main actor together, alongside information programs (i.e., letter, information on social media), that can spread appropriate information on RZV and recommend the vaccine to patients who are at risk of HZ infection.

The results from this study underscored the insufficient knowledge among cardiac HPs regarding RZV. Moreover, they allowed for an assessment of how knowledge about HZ and its prevention influences attitudes and hence practices, as well as how attitudes toward RZV and vaccinations, in general, can shape the clinical practice of cardiac HPs. There is a crucial need for educational interventions, to enhance their ability to reduce the HZ burden through accurate immunization programs. Vaccination is the most cost-effective strategy to reduce morbidity associated with HZ [3]. Evidence indicates that integrating routine vaccines into immunization programs is a successful public health intervention, improving the quality of life of patients at risk of HZ. All HPs, including cardiac healthcare providers, are obligated to promote patient adherence to immunization programs.

Finally, based on our results, a speculation worthy of attention is the potential barriers to knowledge acquisition among cardiac HPs regarding RZV, for which potential corrective actions could be undertaken. These may include factors related to the professional (e.g., limited awareness about the existence or benefits of the recombinant zoster vaccine, preventing its recommendation; inadequate training opportunities or educational programs that specifically address the recombinant zoster vaccine within the context of cardiac care) and factors related to the overall work organization (e.g., time constraints and lack of reminders to engage in continuous education or training programs) [40,41]. However, identifying and analyzing these factors goes beyond the scope of this study, and more research is needed.

Several potential limitations should be considered when interpreting the findings of this survey. First, relying on self-reported information in the survey may have led to either an overestimation or underestimation of HPs’ actual knowledge, attitudes and practices regarding RZV. However, an online written survey with focused questions was designed to minimize this risk. Second, being a single-center real-world study, the number of HPs who agreed to participate was limited, and potential non-response bias may have been present, as it was challenging to assess the characteristics of HPs who chose not to participate. Furthermore, the sample may not have been representative of the Italian cardiac HP population and thus may limit the generalizability of the findings, providing a snapshot that may not accurately represent the broader population. Thus, this research may provide valuable insights into the knowledge of the specific sample studied but may not accurately reflect the broader trends or variations within the entire Italian cardiac HP population. Additionally, the small sample size may not fully mirror the diversity and characteristics of all cardiac HPs in Italy, thus limiting the ability to reflect subtle associations between population characteristics and the outcomes of interest. Lastly, it is important to note that this survey was conducted in a single hospital facility in southern Italy, and recommendations might vary across regions, potentially leading to differences in vaccination accessibility and practices. Therefore, the results from our research might reflect region- or institution-specific practices, and their generalizability could be affected by this limitation.

Despite these limitations, this study used a carefully selected sample size. By applying a formula for estimating a single-population proportion—with the assumption of a 95%CI, a margin of error of 5% and an assumed prevalence of 90% of respondents having an accurate level of knowledge about the major risk factor for HZ, in accordance with the literature [23]—a minimum sample size of 139 was calculated [42]. It is, therefore, possible to state that the survey results provide valuable information about the knowledge, attitudes and practices of the HPs regarding RZV in patients with cardiological conditions. In addition, this study provides insights that will support the same investigation to be replicated in other contexts, contributing to the generation of metrics that can be used to enhance HPs’ knowledge and practices regarding HZ vaccination.

## 5. Conclusions

This study highlights current influences on how healthcare workers in cardiology address RZV. The results suggest possible actions to increase HPs’ knowledge and practices, with the goal of improving vaccination adherence and mitigating the impact of HZ. To boost awareness, it is essential to organize training events that focus on RZV and the protective role of HZ vaccination in patients with cardiovascular conditions, as well as in the general population. More specifically, it would be appropriate for healthcare institutions and scientific societies to integrate information about RZV into existing medical education courses for cardiac HPs. Additionally, they should organize multidisciplinary workshops and seminars involving experts in infectious diseases and immunization, who can provide in-depth insights into HZ prevention and its relevance in cardiac settings. Furthermore, aspects of prevention related to the risks of HZ in patients with cardiovascular conditions should be integrated into clinical practice guidelines as a standard part of the patient care process. Finally, there is great potential for improving vaccine literacy among HPs and, consequently, increasing vaccination coverage through integration between clinicians and prevention professionals specifically involved in vaccination management.

## Figures and Tables

**Table 1:** Selected characteristics of the study population (N = 154).

Characteristic	N	Percentage
Gender		
Male	58	37.7
Female	96	62.3
Age ^^^	45.7 ± 11.3	
Educational level		
Master’s degree or higher	62	40.3
Other	92	59.7
Professional role		
Nurse	99	64.3
Physician	55	35.7
Hospital unit		
Cardiology	18	12.0
Interventional cardiology	23	15.3
Electrophysiology	10	6.7
Cardiac surgery	21	14.0
Post-surgery intensive care	18	12.0
Cardiac intensive care	15	10.0
Cardio-pulmonology	14	9.3
More than one unit	16	10.7
Others/non-cardiology unit	15	10.0
Previous professional update on vaccination		
Yes	61	41.2
No	87	58.8
Experience in managing patients with herpes zoster		
Yes	59	38.3
No	95	61.7

^^^ Expressed as mean and standard deviation.

**Table 2:** Frequency of responses regarding knowledge, attitudes and practices towards recombinant zoster vaccine (RZV).

Item *	N	Percentage
HZ risk factors		
Age > 50 years		
Yes	59	38.3
No	95	61.7
Immunosuppression		
Yes	116	75.3
No	38	24.7
Malignancy		
Yes	33	21.4
No	121	78.6
Multiple chronic conditions		
Yes	57	37.0
No	97	63.0
Major HZ consequences and complications		
Post-herpetic neuralgia		
Yes	82	53.2
No	72	46.8
Recurrence		
Yes	65	42.2
No	89	57.8
Major cardiovascular events		
Yes	55	35.7
No	99	64.3
Knowledge that HZ vaccines are included in the National Vaccination Plan		
Yes	16	10.9
No	131	89.1
Knowledge that zoster vaccine live (ZVL) uses a one-dose schedule		
Yes	72	48.0
No	78	72.0
Knowledge of RZV vaccination schedule (i.e., two doses and timing)		
Yes	43	29.1
No	105	70.9
Knowledge about the possibility of RZV administration in immunocompromised individuals		
Yes	87	57.6
No	64	42.4
Attitude toward RZV safety ^^^	7.4 ± 2.2	
Attitude toward RZV effectiveness ^^^	7.4 ± 2.1	
Have you previously recommended or suggested vaccination to a patient at risk of HZ (either ZVL or RZV)?		
Yes	69	46.3
No	80	53.7
Will you recommend or suggest HZ vaccination to your patients?		
Yes	116	78.9
No	31	21.1

* Numbers for some items may not add up to total number of study population due to missing values. ^^^ Valued on a scale from 1 to 10; results are expressed as mean and standard deviation.

**Table 3:** Multivariate regression models predicting the knowledge, attitude and practice towards recombinant zoster vaccine (RZV).

**Model 1: Knowledge about the possibility of RZV administration in immunocompromised individuals (N = 137)**				
**Variable**	**Odds ratio**	**SE**	**95%CI**	***p*-value**
Log likelihood = −67.68; *χ*^2^ = 45.53 (5 df); *p*-value < 0.0001				
Having a master’s degree or higher	4.42	2.15	1.70–11.47	0.002
Professional update on vaccinations	3.86	1.85	1.51–9.87	0.005
Awareness that HZ could cause major CV events	4.23	2.23	1.50–11.91	0.006
Awareness that HZ could cause PHN	2.56	1.17	1.05–6.25	0.04
Needing further information about RZV	6.43	4.94	1.42–29.98	0.02
**Model 2: Attitude toward RZV effectiveness (N = 132)**				
**Variable**	**Coefficient**	**SE**	**95%CI**	***p*-value**
*F* (5,126) = 42.67; R^2^ = 0.63; adjusted R^2^ = 0.61; *p*-value < 0.0001				
Positive attitude toward RZV safety (≥8 vs. <8/10)	2.92	0.22	2.49–3.36	<0.001
Age (continuous, in years)	−0.02	0.01	−0.04–−0.01	0.02
Experience in managing patients with HZ	0.45	0.21	0.03–0.88	0.04
Awareness that HZ could recur	0.33	0.22	−0.09–0.76	0.13
Awareness that HZ could cause major CV events	0.45	0.22	−0.70–0.15	0.20
**Model 3: Willingness to recommend/suggest HZ vaccination to patients (N = 117)**				
**Variable**	**Odds ratio**	**SE**	**95%CI**	***p*-value**
Log likelihood = −35.92; *χ*^2^ = 31.97 (4 df); *p*-value < 0.0001				
Positive attitude toward RZV effectiveness (≥8 vs. <8/10)	7.17	5.24	1.71–30.03	0.007
Having a master’s degree or higher	7.21	5.92	1.44–36.08	0.02
Age (continuous, in years)	0.94	0.03	0.89–0.99	0.04
Having recommended HZ vaccine in the past	4.03	2.70	1.08–14.96	0.04

Abbreviations: 95%CI, 95% confidence interval; SE, standard error; df, degrees of freedom; RZV, recombinant zoster vaccine; HZ, herpes zoster; CV, cardiovascular; PHN, post-herpetic neuralgia.

## Data Availability

Data and supporting materials associated with this study will be provided upon request by contacting the corresponding author.
